# The mediating effect of social support on uncertainty in illness and quality of life of female cancer survivors: a cross-sectional study

**DOI:** 10.1186/s12955-020-01392-2

**Published:** 2020-05-19

**Authors:** Insook Lee, Changseung Park

**Affiliations:** 1 Department of Nursing, Changwon National University, C.P.O. Box 51140, Changwon, Republic of South Korea; 2 Division of Nursing, Cheju Halla University, Jeju, Republic of South Korea

## Background

Cancer survivors have been defined as those living more than 5 years after cancer treatment with no signs of recurrence or further growth; however, the National Coalition for Cancer Survivorship of the United States defined cancer survivors as those undergoing treatment after being diagnosed with cancer or those considered to be fully cured. The National Cancer Institute of the United States established the Office of Cancer Survivorship, with the American Society of Clinical Oncology including “patient and survivor management” as its 2006 annual objective [1], indicating the importance of cancer survivor management as a major agenda item.

Typically, breast and thyroid cancer diagnoses occur among women in their 40s and 50s, and patients who receive treatment have high survival rates. The majority of breast and thyroid cancer survivors return to their daily lives within a relatively short timeframe [2], making quality of life after treatment and important factor in cancer treatment [3–5].

Cancer survivors have reported experiencing a variety of physical difficulties during or after treatment, including fatigue, pain, loss of energy, sleeping disorders, and constipation [6, 7]. They also have psychological concerns, such as fear of the cancer spreading, concerns about treatment results, and uncertainty about the future [8], as well as financial difficulties, issues with their sex lives, decreased body image, difficulties in interpersonal relationships, role disorders, and difficulty returning to work [7]. Thus, cancer patients require a diverse range of healthcare services, plus emotional and socioeconomic support, with an international study of the quality of life and symptoms of cancer survivors reporting that the quality of life among Asian patients to be the lowest of those than other country [6]. Survivors of breast cancer have reported low quality of life after treatment [9, 10], which is influenced by emotional and psychological factors such as uncertainty, body image, lack of self-respect, and depression [9–11], as well as social factors including social, family, and spouse support [10, 11].

Uncertainty among women diagnosed with malignant illnesses was found to be higher than that among women with a lump in the breast [12]. Uncertainty among breast cancer patients continues for a long period because of the fear of recurrence [13] and reduced quality of life [11]. Similarly, thyroid cancer survivors also show higher levels of fatigue, depression, and anxiety compared to those with no experience of cancer [14–16].

Social support is a complex and multidimensional concept that is characterized by mutual benefits that include social, psychological, and material support provided by the social support network [17]. In other words, social support means help provided by social relationships such as family, friends, and significant others, and plays an important role in directly and indirectly reducing uncertainty [18, 19]. For cancer survivors, the need for social support is varied and depends largely on the adaptive tasks they face [20]. Social support is closely related to breast cancer survivor prognosis [21]; breast cancer survivors’ uncertainty was found to lower their quality of life, but their recognition of social support was found to improve it [11]. Recognition of social support and uncertainty played a key role in managing and maintaining quality of life. Research has shown that social support differs according to survival stage, as patients who are undergoing treatment receive active support from healthcare professionals and their family, but this support declines notably after the treatment ends [14, 22, 23].

With the number of cancer survivors steadily increasing, there has been an increase in the number of studies published on cancer survivors internationally [22, 24]. In Korea in particular, there have been studies on recurrence-prevention behaviors and quality of life [25], the factors influencing quality of life [10], fatigue and quality of life [26], distress and quality of life [27], and symptoms and quality of life among breast cancer survivors [8]. Most of these studies focused on breast cancer survivors, but few studies focus on overall quality of life and the related influential factors. Few studies have analyzed the relationships between uncertainty in illness, quality of life, and social support among female breast and thyroid cancer survivors.

## Methods

### Aims

**Fig. 1:**
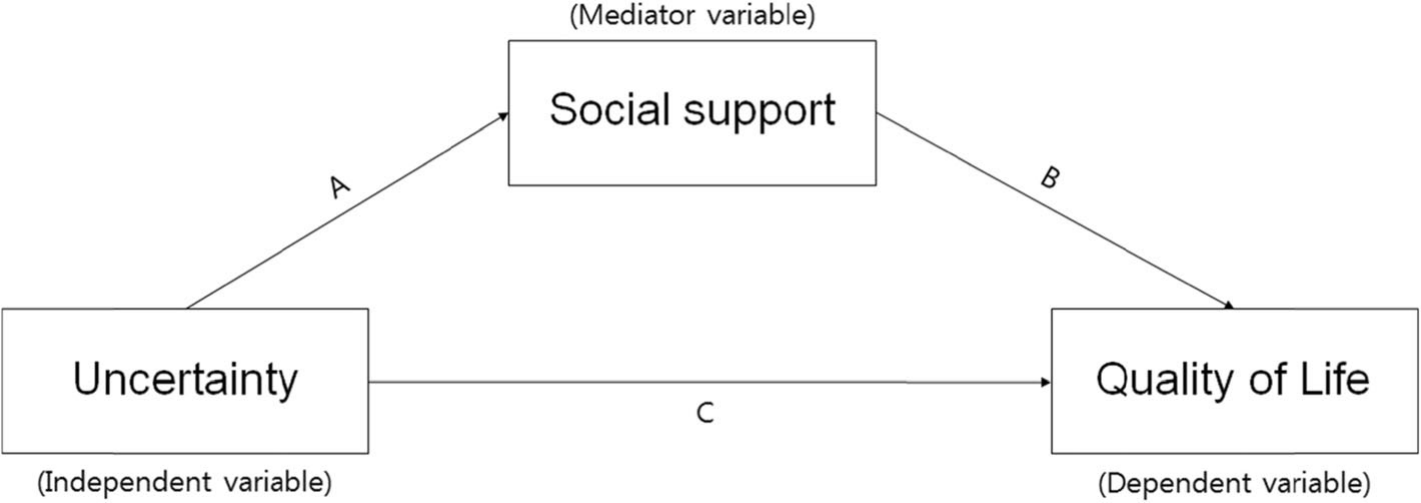
The theoretical research model showing the influence of uncertainty on quality of life and the mediating effect of social support

This study aimed to identify the relationships among uncertainty in illness, social support, and quality of life in female cancer survivors, and to verify the mediating role of social support, in the relationship between uncertainty in illness and quality of life. Social support may act as a generative mechanism influencing how uncertainty in illness, the predictor variable, affects quality of life, the outcome variable (Fig. 1) [28]. Therefore, this study will provide foundational data for devising practical and helpful intervention strategies to raise the quality of life of cancer survivors.

### Participants

Participants were selected using convenience sampling of female cancer patients who were being treated by specialists in breast endocrinology at general hospitals located in J City of Korea. Among the 189 women (138 thyroid and 50 breast cancer patients) who agreed to participate, 156 surveys were collected (response rate: 82.5%). The final sample including 148 participants after excluding eight insincere responses. The data collection period was from April 21 to June 30, 2014. The completion of data collection through the mailed-in copies of surveys occurred on October 15, 2014. Participants were asked to complete the survey, put it in an opaque envelope, and seal it before returning it to the researchers. In cases in which on-site survey completion was difficult, participants were able to complete the survey at home and returned it by mail to the researcher.

The necessary sample size for the multiple regression analysis was confirmed utilizing G*power ver. 3.1.9 with a significance level (α) of .05; power of .80; effect size (f^2^) of .15 (representing a medium effect size in the multiple regression analysis); and 13 independent variables (age, marital status, religion, level of education, occupation, satisfaction with economic status, smoking, drinking, diagnosis name, clinical stage of cancer, time passed since the end of treatment, uncertainty, and social support). The minimum sample size was determined to be 131. Since a maximum dropout rate of 40% was expected, information was collected from a total of 189 participants who fit the following inclusion criteria: 1) a diagnosis of cancer and no cognitive limitations; 2) the ability to understand and complete the survey in Korean; and 3) an understanding of the purpose of the study and consenting to participate. The exclusion criteria were those suffering from a mental illness, those with difficulties in communication, and those who did not wish to participate in the study.

### Measures

#### Uncertainty in illness

Uncertainty occurs when an appropriate subjective interpretation of an illness or event is not formed. This study measured uncertainty using Mishel’s Uncertainty in Illness Scale (MUIS), which is composed of 33 items concerning uncertainty in illness. Mishel [19] originally developed the scale, and it has been translated into Korean by Lee [28]. The MUIS is a self-administered survey, with items scored on a 5-point scale from 5 (*strongly agree*) to 1 (*strongly disagree*). Positive items were measured backward, so that total scores ranged from 33 to 165. Higher scores indicated higher rates of uncertainty. The Cronbach’s α for the original 33-item tool was .91–.93; the Cronbach’s α for the tool used in the study of Korean breast cancer patients [29] was .83. In this study, the Cronbach’s α for the uncertainty scale was .88.

#### Social support

Social support was measured using Zimet et al.’s [30] Multidimensional Scale of Perceived Social Support (MSPSS). This 12-item measure is scored on a 7-point, Likert-type scale, and assessed the three dimensions of family, friends, and significant others. Its sub-domains are composed of four items. Overall social support scores are calculated by summing the scores for each item, with higher scores indicating higher levels of social support. At the time of development, the Cronbach’s α reliability was .91; Cronbach’s α for each subscale ranged from .90–.95. In this study, the Cronbach’s α of the social support scale was .95.

#### Quality of life

Quality of life was measured using a standardized tool that was translated into the Korean and verified validity of the Korean version of the EORTC QLQ-C30, which was developed through a process of international joint study from multiple countries, and it is the most widely used standardized tool to measure the quality of life of cancer patients. This tool is composed of three subdomains and 30 items. It includes two items on overall quality of life and five functional domains (i.e., physical, role, cognitive, emotional, and social functions) that include 15 items; three symptom domains (i.e., fatigue, pain, nausea/vomiting) that include seven items; and one item for each of the symptoms commonly reported by cancer patients (i.e., difficulties in breathing, loss of appetite, sleeping disorders, constipation, diarrhea, and financial hardship) [31]. The EORTC QLQ-C30 is converted into a score ranging between 0 and 100 points [31]; higher overall quality of life scores, higher functional domain scores, and lower symptom domain scores indicate higher quality of life. Moreover, overall quality of life can be understood as a measurement of comprehensive quality of life [31]. This study assess quality of life using the overall quality of life score. At the time of development, Cronbach’s α was .65–.73; the Cronbach’s α for the overall quality of life score in this study was .853.

### Ethical considerations

This study was conducted after receiving approval of the research protocol from the Institutional Review Board (Approved number: 2014-L02–01). The purpose and method of the research was explained directly to participants by a trained research assistant. The participants then signed an informed consent form that stated the survey would be used for the purposes of the study only, and that their confidentiality would be safeguarded. The subjects who agreed to participate in the survey received a small amount of goods worth of KRW 3000, but there were no factors that could interfere with the answers in the survey.

### Data analysis

The collected data were coded and analyzed using SPSS software (version 24.0; SPSS Inc., Chicago, IL) at .05 significance level. The analysis excluded missing data values. The general characteristics, illness-related characteristics, uncertainty, social support, and quality of life were measured using frequency, percentages, means, and standard deviations. The differences in uncertainty, social support, and quality of life in accordance with general and illness-related characteristics were analyzed using independent *t*-tests and one-way analysis of variance (ANOVA). Tukey’s post-hoc analysis was used for independent variables of more than three groups to identify which group contained the differences. Pearson’s correlation coefficient was calculated to identify correlations between uncertainty, social support, and quality of life. Multiple regression (stepwise method) was used to test the influence of uncertainty on social support and quality of life. To verify the mediating effects of social support in the relationship between uncertainty and quality of life, simple, and hierarchical multiple regression analyses were conducted as per the method proposed by Baron and Kenny [30]. The significance of the mediating effects of social support was verified using the Sobel test.

## Results

### Demographic characteristics of subjects

The general characteristics of the female cancer survivors in this study indicated that their average age was 51.87 (*SD* = 11.78) years, with the largest proportion (33.3%) of the population being in their 50s, followed by those in their 40s (30.1%), 60s and over (24.4%), and below 30 (12.2%). Most participants (76.2%) were married; 61.9% practiced a religion, and 66.7% had a high school education or below; 56.2% had jobs; 17.8% had lost their jobs as a result of their cancer diagnosis and treatment, and 73.1% indicated that their satisfaction with their financial status was average. They had an average of 2.26 (*SD* = 1.19) children; 94.4% were non-smokers; 64.1% were non-drinkers; 29.1% of the subjects had breast cancer and 70.9% had thyroid cancer; 53.8% of the cancers were early stage, and 46.2% were advanced. The duration after cancer treatment averaged 17.64 (*SD* = 31.30) months, with 70.3% reporting a duration of less than a year since they ended their treatment (Table 1).

**Table 1:** Participants’ demographic and illness-related characteristics. *N* = 148

Variable	Category	Frequency	%
Age (years)	Below 40	15	12.2
	40–49	37	30.1
	50–59	41	33.3
	60 and over	30	24.4
Marital status	Married	7	4.8
	Single	112	76.2
	Widowed or divorced	28	19.0
Education	Less than high school	92	66.7
	University and higher	46	33.3
Religion	No	56	38.1
	Yes	91	61.9
Having a job	No	38	26.0
	Loss of job	26	17.8
	Yes	82	56.2
Satisfaction with economic status	Satisfaction	9	6.2
	Average	106	73.1
	Dissatisfaction	30	20.7
Number of children	No or 1	27	18.4
	2	70	47.6
	3 and over	50	34.0
Smoking status	Current smoker	8	5.6
	Non-smoker	135	94.4
Drinking status	Current drinker	52	35.9
	Non-drinker	93	64.1
Type of cancer	Breast cancer	43	29.1
	Thyroid cancer	105	70.9
Stage of cancer	Early-stage cancer	71	53.8
	Advanced stage cancer	61	46.2
Time since completion of cancer treatment (years)	Less than 1	104	70.3
	1–5	32	21.6
	5 or more	12	8.1

### Uncertainty in illness, social support, and quality of life

Average scores of uncertainty in illness, social support, and quality of life are given in Table 2. The average uncertainty in illness score was 83.06 (*SD* = 15.29; range: 44–127 points), and the average quality of life score was 66.90 (*SD* = 20.32; range: 0–100 points). Average social support score was 62.62 (*SD* = 17.09; range: 12–84 points), with family support being the highest (mean = 21.84, SD = 6.58), followed by support from significant others (mean = 21.28, *SD* = 5.93) and friends (mean = 19.45, *SD* = 6.70).

**Table 2:** Descriptive statistics for uncertainty in illness, social support, and quality of life. *N* = 148

Variable category		Mean	*SD*	Possible range	Observed range
Uncertainty in illness		83.00	15.29	33–165	44–127
Social support	Family	21.84	6.58	4–28	4–28
	Friends	19.45	6.70	4–28	4–28
	Significant others	21.28	5.93	4–28	4–28
	Total	62.62	17.09	12–84	12–84
QoL	General QoL	66.90	20.32	0–00	0–100

Notes*. SD,* standard deviation, *QoL* Quality of Life

### Differences in uncertainty in illness, social support, and quality of life according to general and illness-related characteristics

The results of the analysis of differences in uncertainty in illness, social support, and quality of life according to general and illness-related characteristics are given in Tables 3 and 4. There were significant differences in uncertainty in illness by educational level (*t* = 4.048, *p* < .001), satisfaction with financial status (*F* = 3.760, *p* = .027), and smoking (*t* = 2.195, *p* = .030). Uncertainty in illness was higher for subjects with less than a high school education, compared to those who had a university degree or higher, when they were dissatisfied with their financial status. Likewise, it was higher for smokers, compared to non-smokers.

**Table 3:** Differences in uncertainty in illness and social support according to demographic and illness-related characteristics. (*N* = 148)

Variable	Category	uncertainty in illness			social support		
		Mean	***SD***	***t*** or ***F*** ***(p-*** value)	Mean	***SD***	***t*** or ***F*** ***(p-***value)
Age (years)	Below 40	77.54	15.10	1.696	66.27	14.14	0.955
	40–49	78.62	15.70	(.174)	64.87	17.26	(.416)
	50–59	84.62	15.61		59.13	18.83	
	60 and over	86.71	15.08		61.45	18.46	
Marital status	Married	66.75	13.94	2.479	72.43	10.97	1.597
	Single	83.91	15.52	(.089)	62.63	17.07	(.206)
	Widowed or divorced	82.68	13.06		59.61	17.92	
Education	Less than high school	86.87	13.87	4.048	62.73	17.19	−0.207
	University and higher	75.16	14.67	(< .001)	63.37	16.43	(.836)
Religion	No	82.08	14.98	−0.387	62.75	19.19	0.060
	Yes	83.28	15.45	(.699)	62.57	15.83	(.952)
Having a job	No	86.45	14.99	1.213	62.22	14.70	0.304
	Loss of job	84.22	12.31	(.301)	65.12	14.85	(.738)
	Yes	81.17	16.29		62.20	18.85	
Satisfaction with economic status	Satisfaction ^a^	72.38	15.57	3.760	66.56	13.88	5.151
	Average ^b^	82.65	14.68	(.027)	64.94	15.80	(.007)
	Dissatisfaction ^c^	89.10	15.95	a < c	54.07	20.39	b > c
Number of children	No or 1	81.00	20.06	0.408	62.19	20.02	0.056
	2	84.45	13.24	(.666)	62.49	16.89	(.945)
	3 and over	82.31	15.40		63.38	14.49	
Smoking status	Current smoker	96.80	20.03	2.195	48.63	27.02	−1.540
	Non-smoker	81.77	14.70	(.030)	63.50	16.29	(.166)
Drinking status	Current drinker	84.95	16.30	1.301	63.78	18.00	0.591
	Non-drinker	81.03	14.30	(.196)	62.00	16.90	(.556)
Type of cancer	Breast cancer	82.71	15.94	−0.140	66.77	14.09	1.911
	Thyroid cancer	83.19	15.16	(.889)	60.89	17.97	(.058)
Stage of cancer	Early-stage cancer	82.76	14.26	−0.490	64.13	13.66	0.241
	Advanced stage cancer	84.28	16.28	(.625)	63.43	18.44	(.810)
Time since completion of cancer treatment (years)	Less than 1	81.95	15.41	1.033	63.45	15.86	4.292
	1–5	87.13	14.02	(.360)	65.00	15.40	(.015)
	5 or more	82.22	17.36		49.25	25.56	a, b > c

Note*. SD* Standard Deviation

Post-Hoc test: Tukey’s test

**Table 4:** Differences in quality of life according to demographic and illness-related characteristics. (*N* = 148)

Variable	Category	quality of life			
		Mean	SD	t or F	***p-*** value
Age (years)	Below 40	67.78	16.92	0.256	.857
	40–49	68.02	20.27		
	50–59	65.85	17.56		
	60 and over	64.08	22.28		
Marital status	Married	66.67	19.25	0.170	.844
	Single	67.87	20.75		
	Widowed or divorced	65.43	14.75		
Education	Less than high school	68.06	20.31	0.563	.574
	University and higher	65.94	21.50		
Religion	No	64.82	20.65	−0.896	.372
	Yes	67.95	20.18		
Having a job	No	65.35	21.79	1.845	.162
	Loss of job	61.33	22.55		
	Yes	69.65	18.22		
Satisfaction with economic status	Satisfaction ^a^	78.70	17.73	6.648	.002
	Average ^b^	69.29	19.38		a, b > c
	Dissatisfaction ^c^	56.32	20.61		
Number of children	No or 1	60.80	21.66	1.726	.182
	2	68.24	19.82		
	3 and over	69.22	19.26		
Smoking status	Current smoker	57.29	32.26	−0.906	.394
	Non-smoker	67.73	19.28		
Drinking status	Current drinker	67.00	20.88	0.015	.988
	Non-drinker	66.94	20.28		
Type of cancer	Breast cancer	69.84	20.16	0.114	.267
	Thyroid cancer	65.71	20.36		
Stage of cancer	Early-stage cancer	68.33	18.91	0.822	.413
	Advanced stage cancer	65.28	23.48		
Time since completion of cancer treatment (years)	Less than 1	67.72	21.91	0.307	.736
	1–5	64.52	16.94		
	5 or more	65.97	13.51		

Note*. SD* Standard Deviation

Post-Hoc test: Tukey’s test

Social support had statistically significant differences given satisfaction with financial status (*F* = 5.151, *p* = .007) and duration since cancer treatment completion (*F* = 4.292, *p* = .015). Social support was higher for subjects with average financial status satisfaction, and for subjects for whom it had been less than a year, or between 1 to 5 years, since they completed cancer treatment.

Quality of life significantly differed according to financial status satisfaction (*F* = 6.648, *p* = .002). Participants had higher quality of life when they had high or average financial status satisfaction compared to dissatisfaction.

### Correlation between uncertainty in illness, social support, and quality of life

The results of the correlation analyses indicated that uncertainty in illness had a significant negative correlation with social support (*r* = −.335, *p* < .001) and quality of life (*r* = −.312, *p* < .001); social support had a significant positive correlation with quality of life (*r* = .321, *p* < .001). Correlations between the sub-factors of social support and quality of life indicate that there were significant positive correlations between quality of life and support from significant others (*r* = .315, *p* < .001), friends (*r* = .284, *p* = .001), and family (*r* = .265, *p* = .001). Uncertainty and support from significant others (*r* = −.326, *p* = .001), friends (*r* = −.294, *p* = .002), and family (*r* = −.244, *p* = .010) showed significant negative correlations; particularly, the highest correlation was between support from significant others and uncertainty (Table 5).

**Table 5:** Correlations among uncertainty, social support, and quality of life. *N* = 148

Variables	Uncertainty in illness		Quality of life	
	***r***	***p-*** value	***r***	***p-*** value
Uncertainty in illness	–	–	−.312	.001
Social support (Total)	−.335	< .001	.321	<.001
Family	−.244	.010	.265	.001
Friends	−.294	.002	.284	.001
Significant others	−.326	.001	.315	<.001

### Mediating effect of social support

Four stages of regression analysis were conducted to verify whether social support had mediating effects in the process by which uncertainty in illness influenced quality of life. Prior to verifying the mediating effects of social support, this study examined the multicollinearity between variables. The residual limit was between 0.8–1.0, which is higher than 0.1; and the value of the variance inflation factor was between 1.0–1.2, which was lower than 10, indicating no issues with multicollinearity. Moreover, the Durbin-Watson test, which is the test of independence of residual error, indicated d = 1.903–1.944, which was close to two and met the independence condition, representing no issues with self-correlation.

**Fig. 2:**
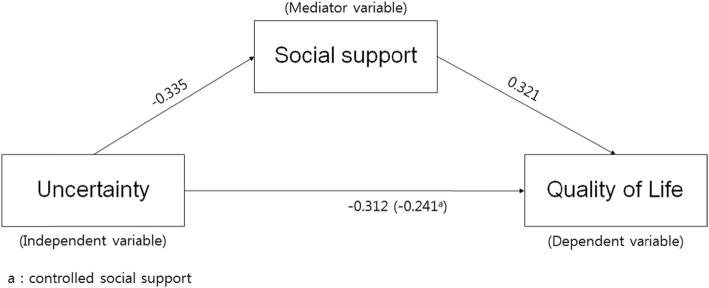
Model showing the influence of uncertainty on quality of life and the mediating effect of social support

Using the hierarchy regression, this confirmed the partial mediating effects of social support in the process of uncertainty influencing quality of life (Table 6, Fig. 2). The first regression analysis indicated that the independent variable (uncertainty) had a statistically significant influence on the mediator variable (social support; β = − 0.335, *p* < .001), and the explanatory power for social support was 10.4%. The second stage regression analysis indicated that the mediator variable (social support) had a significant influence on the dependent variable (quality of life; β = 0.321, *p* < .001), and the explanatory power for quality of life was 9.7%. The third stage regression analysis indicated that the independent variable (uncertainty) had a significant influence on the dependent variable (quality of life; β = − 0.312, *p* = .001) with an explanatory power of 8.9%. At the fourth stage, this study aimed to test the influence of the independent variable (uncertainty) on the dependent variable (quality of life) with social support as the mediator variable. The results indicated that uncertainty (β = − 0.241, *p* = .014) and social support (β = 0.213, *p* = .030) were significant predictors of quality of life. When social support was set as the mediator variable, uncertainty was found to have a significant influence on quality of life; the unstandardized regression coefficient reduced from − 0.396 to − 0.398, indicating a partial mediation of social support. The explanatory power of these variables in terms of quality of life was 12.1%. This study executed the Sobel test to verify the significance of the mediating effects of social support, confirming that they were significant in the relationship between uncertainty and quality of life.

**Table 6:** Mediating effect of social support in the relationship between uncertainty and quality of life. *N* = 148

Equations	*B*	*SE*	β	*t*	*p-*value	Adj. R^2^	*F*	*p-* value
1. Uncertainty → Social support	−0.359	0.098	−0.335	−3.658	< .001	.104	13.380	< .001
2. Social support → QoL	0.383	0.095	0.321	4.044	< .001	.097	16.351	< .001
3. Uncertainty → QoL	−0.396	0.117	−0.312	−3.379	.001	.089	11.417	.001
4. Uncertainty, Social support → QoL						.121	8.322	<. 001
1) Uncertainty → QoL	−0.308	0.123	−0.241	−2.500	.014			
2) Social support → QoL	0.257	0.116	0.213	2.206	.030			
Sobel test: Z = −2.711, *p* = .007								

*Note. QoL* Quality of Life

## Discussion

Mullen divided the stages of cancer survival into three major classifications [32, 33]. First is the acute stage, which marks the period after the cancer diagnosis. Second is the extended stage, in which the active treatment of cancer has ended and the patient is placed under tracking observation or engages in intermittent treatment. During this period, the majority of cancer survivors experience uncertainty toward their cancer treatment and fear recurrence, and they may experience physical and psychological issues. Lastly, the permanent stage marks a period in which the cancer is thought to be fully cured, or the patient is expected to survive long term, with a low risk of recurrence.

The participants in this study averaged a score of 66.90 for quality of life. As it is difficult to draw a direct comparison given the lack of research utilizing this measure, in converting quality of life into a scale of 100 points, this study’s results were similar to those found previously regarding post-hoc management following breast cancer treatment for 200 women [8, 10]. However, a study covering regionally based, adult female breast cancer survivors between 6 months and 2 years after anti-cancer treatment completion reported lower scores (e.g., 60.13 points) compared to this study [27]. Likewise, a study of breast cancer survivors with completed surgeries and assistive treatments, breast cancer survivors whose treatment had ended had scores of 53.4 and 56.66 points, respectively for breast cancer survivors with completed surgeries and assistive treatments [25, 26].

On the other hand, a report of 110 adult females with breast cancer or OB/GYN cancers [33] indicated that quality of life according to cancer survival stage was 58.7, 62.3, and 66.8 points during the acute, extended, and permanent stages, respectively. Quality of life in this study was similar to the level experienced by survivors during the permanent stage. Considering that the average time since treatment was 17.64 months, these results indicate a relatively high quality of life. While these differences cannot be accurately compared and discussed because of the lack of research covering the same variables, the majority of survivors had thyroid cancer (70.9%), and it is known that thyroid cancer has higher rates of survival. Going forward, it is important to develop interventions to improve quality of life by assessing survivors’ specific stages.

There were no significant relationships between quality of life and length of time since completing treatment. Existing research has suggested that quality of life was significantly higher for those surviving more than 5 years after cancer treatment completion [8, 33], indicating that quality of life improves as duration of survival increases. The quality of life of these cancer survivors has been reported to improve with the passage of time [8, 33]. Therefore, cross-sectional and longitudinal studies are required in the future to identify quality of life by survival stage and changes in quality of life over time.

On the other hand, qualitative studies of Korean female cancer survivors have indicated that the significant others and families of female cancer survivors wanted them to return to their pre-cancer lives to take care of their spouses and children, indicated the demands on female cancer survivors in Korea to fulfill their roles as wives and mothers before fully recovering from cancer [33]. Thus, customized interventions by survival stage for female cancer survivors are needed along with further research on the relationships between cultural specificity, role conflicts imposed on survivors because they are women, and their quality of life.

The uncertainty toward illness of the participants in this study was similar to existing research in breast cancer patients undergoing chemotherapy averaged 83.08 [34] and female thyroid cancer patients [35]. On the other hand, the level of uncertainty faced by cancer patients prior to surgery averaged 81.43 in a study of cancer patients hospitalized for breast, thyroid, and bladder cancer [36], which was slightly lower than the value found in this study. This appears to be because female cancer survivors in this study were mostly in the extended stage, which comes after the active treatment of their cancer [32, 33]. Most cancer survivors face uncertainty toward cancer treatment and fear of recurrence [8, 32, 33]; thus, they experience a diverse range of physical and psychological problems [6, 7]. On the other hand, a qualitative study of 25 breast cancer survivors aged over 30 who had undergone surgery and chemotherapy as their primary treatment for breast cancer [37] indicated that quality of life following treatment for breast cancer survivors saw a coexistence of anxiety and uncertainty about recurrence. A shorter duration of time since treatment led to higher confusion in their own health management efforts and health management in general.

These results indicate that there are limitations to comparing uncertainty results given the lack of domestic studies on cancer survivors; therefore, future studies are needed to fill this gap. Moreover, it is necessary to confirm uncertainty by cancer survival stage and develop interventions to reduce the uncertainty accompanying each stage.

Uncertainty in illness was higher for those with less than a high school education, compared to those with a university education or higher, when they were dissatisfied with their financial status, and for those who were smokers. These results were similar to previous research [36], which indicated high uncertainty for participants over 60 who had a low monthly income and low level of education. Therefore, it is necessary to consider these socioeconomic factors when developing uncertainty reducing strategies such as customized information delivery and communication.

The social support of female cancer survivors in this study was rather high, at 62.62 out of 84 points; family support was the highest, followed by support from significant others, and finally friends. Social support is known to play an important role in helping individuals reduce their levels of uncertainty [37]. Particularly, in Korea, family and healthcare professional support have been the most important support resources among all social support types [34]. The results of this study indicated that family support was the highest, which was in line with the results of existing studies. On the other hand, a qualitative study of 25 breast cancer survivors aged over 30 who had undergone surgery and chemotherapy as their primary breast cancer treatments [37] indicated that positive support and responses from family, patients with similar illnesses, and those surrounding them helped to strengthen positive self-suggestion, which also helped them to overcome their illnesses. Other studies have reported that patients undergoing treatment receive active support from healthcare professionals and their family, but they receive less support and interest from healthcare professionals, their family, and those surrounding them after the treatment ends [22, 23, 27]. Therefore, it is necessary to take a continuous interest in and facilitate social support for cancer survivors.

Social support was higher for participants with average satisfaction toward their financial status, and for those for whom less than a year, or between 1 and 5 years, had passed since the completion of their cancer treatment, compared to those for whom 5 years or more had passed since treatment. These results were similar to those of studies on cancer patients hospitalized for breast, thyroid, and bladder cancer surgery [36], which indicated social support differed according to the time that had passed since diagnosis. Moreover, these results are similar to those reporting that breast cancer survivors are required by their spouses or family to fulfill roles they had filled prior to their cancer diagnosis, and this was associated with decreasing support from family [38]. These results indicate that female cancer survivors require ongoing psychosocial support as well as education and access to information as they live out their lives.

According to Baik and Lim [20], who studied social support according to different stages of breast and gynecological cancer survival, the social support of patients in the acute stage was comparatively higher, but there were no significant differences in social support across the different stages, which was different from the findings of this study. While there were no significant differences, Baik and Lim [20] reported that the social support perceived by survivors decreased as they proceeded through the acute to the extended stage. The social support perceived by respondents decreased in the 2 years following the diagnosis but maintained the reduced rate through the permanent stage [20]. Long-term survivors had a greater need to meet other cancer patients and self-help groups [20]. Kwon and Yi [27] asserted that interest and support from family and the society in general are very important in raising breast cancer survivors’ quality of life and survival rates. Moreover, self-help groups were reported to be effective in providing emotional support for long- and short-term cancer survivors [39], which indicates the need for developing stage-specific social support interventions and various methods of facilitating social support groups. Moreover, further research is required concerning cancer survival stage-dependent social support and quality of life.

The results of this study showed that higher uncertainty in illness among female cancer survivors led to reduced social support and quality of life, while higher social support led to better quality of life. Support from others was found to be the most relevant aspect of the relationship between quality of life and uncertainty. These results were similar to those of studies on early-stage breast cancer patients [40] and on cancer patients hospitalized for breast, thyroid, and bladder cancer surgery [36], which indicated that perceived social support was lower as uncertainty increased.

Uncertainty was very influential on female cancer survivors’ quality of life. Higher uncertainty in illness among female cancer survivors led to lower social support and reduce quality of life; higher social support led to improved quality of life. The explanatory power of these variables on quality of life was 12.1%; uncertainty in illness and social support influenced the quality of life of female cancer survivors. Moreover, in the process of uncertainty in illness influencing subjects’ quality of life, social support was confirmed to play a significant, partially mediating, role in the relationship between uncertainty and quality of life. Higher uncertainty toward illness led to lower quality of life, higher social support led to higher quality of life, and social support influenced female cancer survivors’ quality of life by partially mediating its relationship with uncertainty. Social support plays an important role in directly and indirectly reducing uncertainty [18, 19]. Social support is closely related to the prognosis of breast cancer survivors [21]. Uncertainty among breast cancer survivors has been found to lower their quality of life; however, social support has been found to improve quality of life [11]. Thus, the need for a diverse range of attempts, including developing and applying social support programs, to increase cancer survivors’ quality of life exists. On the other hand, the partially mediating effects of social support indicate that there are other mediating factors in uncertainty in illness’s influence on quality of life. Therefore, it is important for future studies to include other mediating factors in their examinations of what influences quality of life among female cancer survivors.

In Korea, studies on cancer survivors have been conducted since 2010, and the majority of these focused on breast cancer survivors. Particularly, as there has been no overall research into the healthy behaviors of cancer survivors, it is necessary to develop practical guidelines that befit Korea through studies concerning the development and application of health improvement programs based on the study of healthy behaviors, as per the assertion of Kim [32]. Moreover, attempts are needed to practically apply a diverse range of intervention studies to improve cancer survivors’ quality of life.

Moreover, future studies should include mediator variables other than social support that might influence quality of life. Additionally, both cross-sectional and longitudinal studies are needed to further investigate the quality of life and uncertainty according to the stages of survival.

## Conclusion

Our results show that social support partial mediates the relationship between uncertainty and quality of life in female cancer survivors. The results of this study have great implications for improving cancer care, especially in how it relates to quality of life, and they also demonstrate how uncertainty can be decreased. Therefore, it is necessary to develop and apply intervention methods to improve social support thereby improving quality of life among female cancer survivors. A nurse-led social support program may especially contribute to enhancing the quality of life of cancer survivors by providing them with adequate health information and emotional support.

## Data Availability

The data used in this study were collected through questionnaires and analyzed by coding the original data. The datasets generated and/or analysed during the current study are not publicly available due [REASON WHY DATA ARE NOT PUBLIC] but are available from the corresponding author on reasonable request.

## Abbreviations

MUIS: Mishel’s Uncertainty in Illness Scale
MSPSS: Multidimensional Scale of Perceived Social Support
EORTC QLQ-C30: European Organization for Research and Treatment of Cancer Quality of Life Questionnaire Core 30
OB/GYN: Obstetrics/Gynaecology
